# Bird Communities and Biomass Yields in Potential Bioenergy Grasslands

**DOI:** 10.1371/journal.pone.0109989

**Published:** 2014-10-09

**Authors:** Peter J. Blank, David W. Sample, Carol L. Williams, Monica G. Turner

**Affiliations:** 1 Department of Zoology, University of Wisconsin, Madison, Wisconsin, United States of America; 2 Wisconsin Department of Natural Resources, Madison, Wisconsin, United States of America; 3 Wisconsin Energy Institute, University of Wisconsin, Madison, Wisconsin, United States of America; Ohio University, United States of America

## Abstract

Demand for bioenergy is increasing, but the ecological consequences of bioenergy crop production on working lands remain unresolved. Corn is currently a dominant bioenergy crop, but perennial grasslands could produce renewable bioenergy resources and enhance biodiversity. Grassland bird populations have declined in recent decades and may particularly benefit from perennial grasslands grown for bioenergy. We asked how breeding bird community assemblages, vegetation characteristics, and biomass yields varied among three types of potential bioenergy grassland fields (grass monocultures, grass-dominated fields, and forb-dominated fields), and assessed tradeoffs between grassland biomass production and bird habitat. We also compared the bird communities in grassland fields to nearby cornfields. Cornfields had few birds compared to perennial grassland fields. Ten bird Species of Greatest Conservation Need (SGCN) were observed in perennial grassland fields. Bird species richness and total bird density increased with forb cover and were greater in forb-dominated fields than grass monocultures. SGCN density declined with increasing vertical vegetation density, indicating that tall, dense grassland fields managed for maximum biomass yield would be of lesser value to imperiled grassland bird species. The proportion of grassland habitat within 1 km of study sites was positively associated with bird species richness and the density of total birds and SGCNs, suggesting that grassland bioenergy fields may be more beneficial for grassland birds if they are established near other grassland parcels. Predicted total bird density peaked below maximum biomass yields and predicted SGCN density was negatively related to biomass yields. Our results indicate that perennial grassland fields could produce bioenergy feedstocks while providing bird habitat. Bioenergy grasslands promote agricultural multifunctionality and conservation of biodiversity in working landscapes.

## Introduction

Interest in bioenergy is increasing in the U.S. due to concerns about climate change, energy independence, air and water quality, and other issues [1], [2]. Most biofuels in the U.S. are currently made from corn grain [2], but biofuels derived from cellulose (e.g., cellulosic ethanol) are an important part of the national renewable energy policy [3]. Using perennial grasslands to produce bioenergy feedstocks could help meet national cellulosic bioenergy goals and could promote multifunctionality of working lands by producing agricultural commodities and ecological benefits [4], [5], [6], [7], [8]. However, grassland production for bioenergy is still in its infancy [9], and the degree to which grassland production and conservation goals can be aligned is uncertain. Notably, the influence of potential bioenergy grassland crop types on biodiversity is not well understood.

Among potential bioenergy crops, low-input high-diversity grassland fields, such as native prairies, could provide greater biodiversity value and ecosystem services compared to high-input low-diversity fields, such as intensively managed annual row crops [10], [11], [12]. Grasslands planted with native warm-season (C_4_) grasses, such as switchgrass (*Panicum virgatum*) and big bluestem (*Andropogon gerardii*), have high bioenergy potential [13], [14] and could provide valuable wildlife habitat [7], [15]. Grassland birds, which have experienced substantial population declines in recent decades [16], may particularly benefit if diverse grasslands are used for bioenergy production [10], [17]. Bird use of perennial bioenergy grasslands could be influenced by vegetation characteristics of the fields [18], [19], [20], [21], [22] and by landscape context around the fields [23], [24], [25], [26], [27]. To date, few studies have assessed the value of potential bioenergy grasslands for breeding birds, and among the studies on the potential effects of biomass crops on biodiversity, many lack appropriate study designs and spatial replication, making inferences from these studies limited [28].

In addition to its effects on birds, plant diversity in perennial grasslands may also affect biomass yields and therefore bioenergy potential [7]. Tilman et al. [12] found that greater plant diversity was associated with higher energy yields in experimental plots in Minnesota, but this research has been contradicted by several studies [e.g., 7,29]. For example, greater plant diversity was associated with lower biomass yields in Conservation Reserve Program (CRP) grasslands in the northeast U.S. [30]. Compared to monocultures, diverse plant mixtures have greater variation in chemical content and physical properties that lower bioenergy conversion efficiency and add cost to conversion processes [30], [31]. Because it is unclear if industrial-scale bioenergy production will call for monotypic or mixed-species (i.e., polycultures) production systems [29], [32], more information is needed about biomass productivity in potential grassland bioenergy crops [2]. An improved understanding of the biomass potential of grassland crops will also allow tradeoffs between biomass yields and biodiversity to be evaluated [7], [33].

We studied bird community assemblages in potential bioenergy crops along a gradient of plant diversity ranging from intensively managed cornfields and grass monocultures, to more diverse grass-dominated fields (grasslands with >50% live vegetation in grass), to diverse forb-dominated fields (grasslands with <50% live vegetation in grass). In each grassland field we also measured vegetation characteristics and biomass yields. We asked: (1) How do bird communities differ among cornfields and potential bioenergy grasslands? (2) How do vegetation and landscape context influence bird communities in potential bioenergy grasslands? (3) How do biomass yields differ among potential bioenergy grassland types? (4) What are the tradeoffs between producing biomass and providing bird habitat in perennial grassland fields? A main objective of the study was to develop recommendations for improving the habitat quality of future grassland biomass production fields for grassland birds. This study is one of the few to assess how bird communities are influenced by vegetation characteristics and landscape context of potential grassland bioenergy crops, and the first to quantitatively evaluate the tradeoffs between biomass production and bird habitat in perennial grassland fields.

## Methods

### Study Area

**Figure 1:**
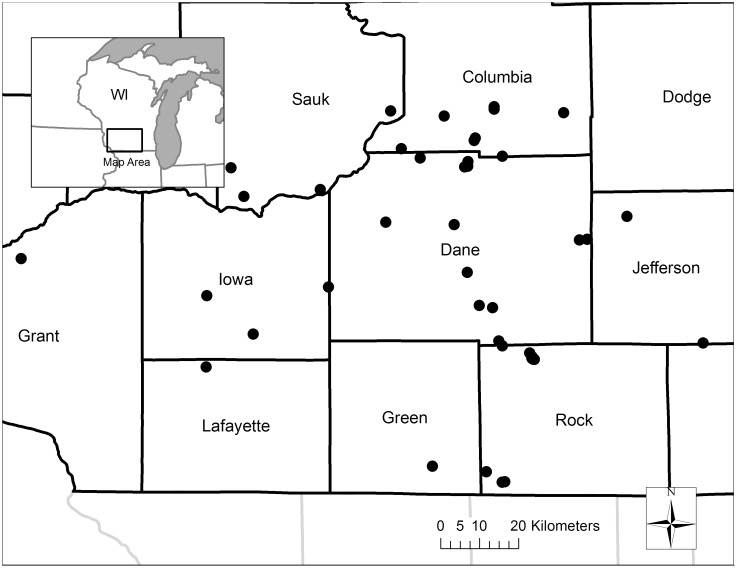
Study sites (solid circles) and counties in southern Wisconsin, USA.

Our study was conducted in southern Wisconsin, U.S. (Fig. 1), a predominantly agricultural landscape representative of much of the Midwest. Climate of the region is continental (warm humid summers and cold winters), with annual precipitation averaging 86 cm and occurring largely from May to September [34]. However, the region experienced a severe drought in the summer and fall of 2012 [35]. Topography is generally flat to rolling, with gentle hills and some shallow depressions containing wetlands. Soils are primarily composed of prairie-derived Mollisols and forest-derived Alfisols [36]. Agricultural lands are dominated by corn, soybean, alfalfa, small grains, and livestock pastures, but the region also includes forests, restored grasslands, lakes, wetlands, and a densely populated urban area (Madison, Wisconsin). Southern Wisconsin is well suited for this study because the central U.S. is predicted to have high local biomass production of switchgrass under future climate scenarios [37], and several grassland bird species nesting in the study area are listed as Species of Greatest Conservation Need (SGCN) in Wisconsin [38].

### Study sites

We established study sites on 30 grassland fields and 11 cornfields (Fig. 1; Tables S1 and S2). At present, there are few grassland fields grown for biomass feedstocks in Wisconsin [9], [39], and most are small agronomic research plots unsuitable for field-scale research on bird communities. Therefore, we used existing perennial grassland fields to represent grasslands that could be harvested for biomass [as in 15,40]. Among the grassland fields, five were grass monocultures managed for seed production and 25 were planted for conservation purposes and were not commercially harvested. Average field sizes were 22 ha for cornfields (SE = 7 ha) and 19 ha for grassland fields (SE = 3 ha). All of the grassland fields we studied were planted with warm-season grasses because these grasses are considered to have high bioenergy potential [13], [14], [30]. For example, switchgrass is considered a valuable bioenergy feedstock source because of its perennial growth, relatively easy establishment, ability to grow on marginal agricultural lands, low nutrient requirements, high yield potential, and drought resistance [13], [41]. We only included grassland fields that had no significant woody cover because we assumed that bioenergy production fields harvested annually would have little to no woody cover.

The five grass monocultures were identified after a thorough search for commercial-scale grass monocultures in Wisconsin. The grass monoculture fields were intensively-managed, seed production fields in which seeds are harvested in autumn and later sold for native grass plantings. They included one switchgrass field, two big-bluestem fields, one indiangrass (*Sorghastrum nutans*) field, and one field with switchgrass on one side of the field and indiangrass on the other (the two grasses on this field were not grown as a mixture, therefore we considered it a monoculture). Three of these grass monocultures, owned by Agrecol Corporation in Evansville WI, are currently being used to produce bioenergy feedstocks: the seed waste (hulls and straw) from their seed cleaning operation is used to produce grass biomass pellets that are burned to heat their production facilities and have been sold for residential and commercial heating [42]. To our knowledge, our study is the first analysis of the potential effects of grassland bioenergy production on bird communities to include study fields that are actually used to produce biomass feedstocks for bioenergy.

We classified the 25 conservation grassland fields as either grass-dominated (>50% of live vegetation cover in grass; *n* = 14) or forb-dominated (<50% of live vegetation cover in grass; *n* = 11), in part based on Garlock et al. [31] who classified cellulosic ethanol feedstocks into grass- and forb-dominated samples. Switchgrass, indiangrass, and big bluestem were the most common grasses among grassland sites (Table S1). Commonly encountered forbs among grass- and forb-dominated fields included wild bergamot (*Monarda fistulosa*), pinnate prairie coneflower (*Ratibida pinnata*), blackeyed susan (*Rudbeckia hirta*), Canada goldenrod (*Solidago canadensis*), wholeleaf rosinweed (*Silphium integrifolium*), and compassplant (*Silphium laciniatum*; Table S1). Thus, the grass- and forb-dominated grassland fields represent a range of grass-to-forb cover ratios that may be harvested for bioenergy in the future if cellulosic feedstock sources other than grass monocultures are desired.

Study sites were located on private farms, nonprofit conservation areas, U.S. Fish and Wildlife Service Waterfowl Production Areas (WPAs), and Wisconsin Department of Natural Resources (DNR) wildlife areas. Permission for the research was granted from each of the private farm owners and from the Wisconsin DNR for work on the wildlife areas. The nonprofit conservation areas and the WPAs were open to the public and no permits or approvals were necessary to work on those properties. Three of the grassland fields on private lands were enrolled in the CRP.

### Bird surveys

We surveyed one 100-m radius bird point count circle in each field. The point was randomly located within the field and at least 100 m from the field edge. All points were ≥350 m apart. We surveyed 17 fields in 2011 and 31 fields in 2012. Seven fields were surveyed in both years (one cornfield and six grassland fields). Within each year, fields were surveyed three times between 30 May and 13 July. Each point count was 10 min in duration and all birds seen or heard were recorded (Dataset S1). All surveys were between sunrise and four hours after sunrise and were not conducted during rain, heavy fog, or sustained winds >16 km/hr. One observer conducted each survey. All surveys were conducted by PJB or E.R. Keyel. Because the bird surveys were strictly observational and did not involve bird handling, no animal research or ethical approvals were required to conduct this research.

### Vegetation Surveys

We surveyed vegetation composition and structure in all grassland fields in the same year in which they were surveyed for birds (Dataset S2). In 2011 vegetation surveys were conducted between early and mid-August, and in 2012 surveys were conducted between late-June and mid-July. We established three vegetation transects evenly spaced within each 100-m radius bird point count circle. The middle transect went down the center of the circle and the outer transects averaged 60 m from the middle transect. We surveyed vegetation in four 0.5-m^2^ Daubenmire plots [43] on each transect, totaling 12 plots per circle. Plots were evenly spaced and approximately 44 m apart along each transect. Within each plot we visually estimated the percent cover of total canopy (live + residual vegetation), live grasses (warm- and cool-season), live forbs, and each individual live plant species. We estimated plant species richness by counting the total number of live plant species per plot. Litter depth was measured at three evenly spaced positions in each plot. We measured vertical vegetation density from visual obstruction measurements, taken in the four cardinal directions, of a modified Robel pole when the pole was viewed from a distance of four m and a height of 1.5 m [44]. Average litter depth and vertical density per plot were calculated from the multiple measurements in each plot. Percent of live vegetation in grass was calculated by dividing the percent cover of live grass by the sum of the percent cover of live grass and forbs.

### Landscape analysis

We superimposed the point-count locations onto the 2011 cropland data layer [45] in ArcMap 10 [46]. Land-cover classes were reclassified as cropland, grassland/pasture, forest, wetland, low development, high development, barren land, and shrubland. Grassland and pasture were combined into one category called grassland [as in 47]. We evaluated R^2^ values from univariate models testing the influence of the proportion of agriculture and grassland in 1-, 2-, 3-, 4-, and 5-km radius landscapes around each point on several bird response variables (e.g., total bird density, SGCN density). These results indicated that the 1-km radius landscape scale was equal to or better than other landscape scales at explaining variation in bird responses to landscape factors. Aerial photographs of the study sites also indicated that 1-km radius landscapes around the points encompassed the dominant land-cover types near the study fields. Therefore, we used a 1-km radius landscape scale for further statistical analyses of landscape metrics (Dataset S2), which is a scale that has been related to grassland bird community structure in other studies (e.g., [24]).

### Biomass and Gross Bioenergy Yields

We measured fall biomass yield in October 2012 by recording leaf area index (LAI) and predicting biomass yield with an allometric equation we developed for our study area. We chose October because it is when farmers will harvest biomass according to best management practices that conserve nutrients [33]. In 20 grassland fields, we measured LAI in 12 plots laid out in the same arrangement as the summer vegetation survey plots (see above) with an AccuPAR LP-80 Ceptometer (Decagon Devices, Inc., Pullman, WA). From nine of these fields, aboveground biomass was harvested at 10–15 cm stubble height [13], [30] in four 0.5-m^2^ plots (*n* = 36 harvested plots) chosen to span a range of biomass yields. Biomass was dried at 60°C for at least five days and then weighed. We related harvested aboveground biomass to LAI from 35 of the 36 plots (one plot was omitted due to measurement error):
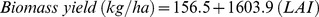
(1)


Based on this equation (*R* ^2^ = 0.72), average fall biomass yields were predicted for all 20 grassland fields on which LAI was measured (Dataset S2).

To predict fall 2012 biomass yield in five grassland fields that were mowed before we could sample LAI and in the 11 grassland fields surveyed in 2011, we developed an equation to relate October 2012 biomass yield to seven vegetation variables measured in summer 2012 [percent cover of canopy (Canopy), total grasses (Grass), warm-season grasses (WSG), cool-season grasses (CSG), and forbs (Forbs); vertical density (Robel; min. height of Robel pole visibility, dm), and litter depth (LD, dm)]:
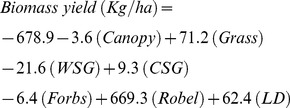
(2)


This equation (*R* ^2^ = 0.72) was used to predict fall biomass yields in the remaining 16 fields (Dataset S2).

### Statistical Analyses

We developed models for three community-level bird metrics (bird species richness, total bird density, and density of SGCNs) and the density of the four most commonly detected bird species. Recently, methods have been developed that incorporate detection probabilities into estimates of species richness and densities of animal populations [e.g., 48,49,50]. However, after evaluating many different model structures that incorporate detection probabilities, we were unable to find models that adequately fit the data. Therefore, we used unadjusted counts as the response variables in our models. We believe that detection rates were relatively high and were consistent among fields because all of our counts were conducted under favorable weather conditions with low wind and no precipitation, surveys were conducted by two skilled observers (PJB and E. R. Keyel), it was very rare to observe birds before or after the counts that were not detected during the counts, and the vegetation was short enough so that most birds detected were observed by sight and sound. However, we recognize that detection may have been <100% [51] and that our counts may be biased low, and as such our counts are indices of abundance. Thus, in this paper we estimate relative and not absolute effects of potential bioenergy crop fields and landscape attributes on bird metrics.

All analyses were performed in R 3.0.1 [52]. For all bird models, we fit generalized linear mixed models with the lmer function in the lme4 package [53], specified a Poisson distribution, and included site and year as random effects. Because there were very few birds in cornfields, we were unable to fit models that included cornfields as a factor level. Differences in bird metrics among grassland field types were tested by specifying field type as a fixed factor and including the percent cover of agriculture and grassland within 1 km as covariates to account for landscape-level effects.

We used an information theoretic approach [54] to evaluate the influence of vegetation characteristics and landscape context on each of the bird metrics. This approach allows for inference from multiple models of the same response variable, produces a weight of evidence for each candidate model, and incorporates model selection uncertainty into parameter estimates. The full (global) model of each bird metric included three vegetation explanatory variables (Forbs, WSG, and Robel) and two landscape explanatory variables at the 1-km radius scale [percent cover of agriculture (Ag) and (Grassland)]. Robel^2^ was also included to test for non-linear relationships with vertical density, because previous research suggests that bioenergy grasslands may be suitable for the widest array of grassland birds at intermediate levels of vegetation density [40]. Among the vegetation variables measured, we did not include plant species richness, grass cover, and total canopy cover because they were highly correlated with other vegetation variables (*r*≥0.7), and did not include litter depth and cool-season grass cover because they had highly skewed distributions. The proportion of forest in the landscape was omitted because it was highly correlated with proportion of agriculture (*r* = −0.7), and we did not include other land-cover classes because they had highly skewed distributions or comprised a small proportion of the study landscapes. We tested all possible combinations of the six retained explanatory variables for each of the bird response variables, including a null model with no covariates. The predictor variables were centered and standardized before entering them into the models to improve the interpretability of the regression coefficients (Schielzeth, 2010). We tested the assumptions of the global models by plotting the residuals against the fitted values and each covariate in the full model to ensure that the residuals did not spread as the fitted and covariate values increased [55]. All of the global models met the assumptions. We also produced model-averaged estimates, and 95% unconditional confidence intervals, of continuous explanatory variables with the modavg function in the AICcmodavg package [56]. If the unconditional confidence intervals of the model-averaged parameter estimates did not overlap zero, we interpreted this as strong support that the predictor variable was related to the response.

Differences in fall biomass yields among grassland field types were also evaluated by using a mixed model with the lmer function, with field type as a fixed factor and site and year as random factors. We evaluated relationships between fall biomass yields and summer vegetation variables with the lme function in the nlme package [57] by using biomass yield as a response variable and each vegetation variable as an explanatory variable in separate models. We predicted bird metrics as a function of Robel, and included Robel^2^ in the prediction models because model-averaged parameter estimates indicated that Robel^2^ was strongly related to several bird metrics. Because Robel was strongly correlated with biomass yield (Biomass), we also predicted bird metrics as a function of Biomass and Biomass^2^. We did not include quadratic terms in the prediction models of SGCN density because there was little evidence that those terms improved the models. Forbs and Grassland were included as covariates and were held constant at their means in the bird prediction models because these variables were found to consistently influence bird response. One grass monoculture field with a high estimated biomass yield in 2011 relative to other fields (12.02 Mg/ha) was omitted from the biomass prediction models because it was a statistical outlier that had a high influence on the results.

## Results

Grasses represented 98%, 66%, and 32% of the live vegetation cover in grass monoculture, grass-dominated, and forb-dominated fields, respectively (Table 1). Litter depth was close to zero in grass monocultures, and vertical density was similar between grass monocultures and forb-dominated fields. Across all grassland fields, forb cover was positively correlated with plant species richness (*r* = 0.8) and negatively correlated with live vegetation in grass (*r* = −0.8). The 1-km radius landscapes around the grassland sites were primarily composed of agriculture (mean = 36%, range: 3 to 64%), grasslands (mean = 30%, range: 7 to 75%), forests (mean = 17%, range: 1 to 58%) and wetlands (mean = 10%, range: 0 to 32%).

**Table 1:** Descriptive statistics of vegetation variables in the three grassland field types^a^.

	Grass monoculture		Grass-dominated		Forb-dominated	
Variable	Mean	SE	Mean	SE	Mean	SE
Plant species richness (# species/0.5 m^2^)	1.5	0.2	5.2	0.5	6.2	0.6
Total canopy cover (%) (live + residual)	54.2	7.8	38.6	3.4	47.9	4.2
Total grass cover (%) (live)	53.5	8.1	21.6	2.1	12.3	1.6
Warm-season grass cover (%) (live)	39.7	8.3	16.2	2.7	16.6	1.8
Cool-season grass cover (%) (live)	1.7	1.7	15.5	2.9	13.5	2.6
Live vegetation in grass (%)	98.4	0.6	66.0	3.5	31.6	3.2
Forbs (%) (live)	0.9	0.3	11.0	1.6	29.1	4.0
Vertical density (min. height of Robel pole visibility, dm)	6.1	1.3	3.6	0.4	6.6	0.5
Litter depth (cm)	0.1	0.1	6.5	1.5	6.7	1.6

a Data are from 11 sites in August 2011 and 25 sites in July 2012. Sample sizes: grass monoculture (*n* = 5), grass-dominated (*n* = 14), forb-dominated (*n* = 11). For six sites measured in both years, the mean was calculated across years.

Twenty-nine bird species were detected among all study sites (Table S3), including six species in cornfields, eight in grass monocultures, 19 in grass-dominated fields, and 20 in forb-dominated fields. Thirteen species were habitat generalists that commonly occur in grasslands, 11 species were grassland obligates, and 10 species are considered SGCNs in Wisconsin. Of the SGCNs, we observed none in cornfields, three in grass monocultures, eight in grass-dominated fields, and seven in forb-dominated fields. Three habitat generalists [Red-winged Blackbirds (*Agelaius phoeniceus*), Song Sparrows (*Melospiza melodia*), and Common Yellowthroats (*Geothlypis trichas*)] and one grassland obligate species [Dickcissel (*Spiza americana*)] had the greatest overall densities and were detected at the most sites. Cornfields and grass monocultures had the lowest bird densities for most species compared to grass- and forb-dominated fields.

**Figure 2:**
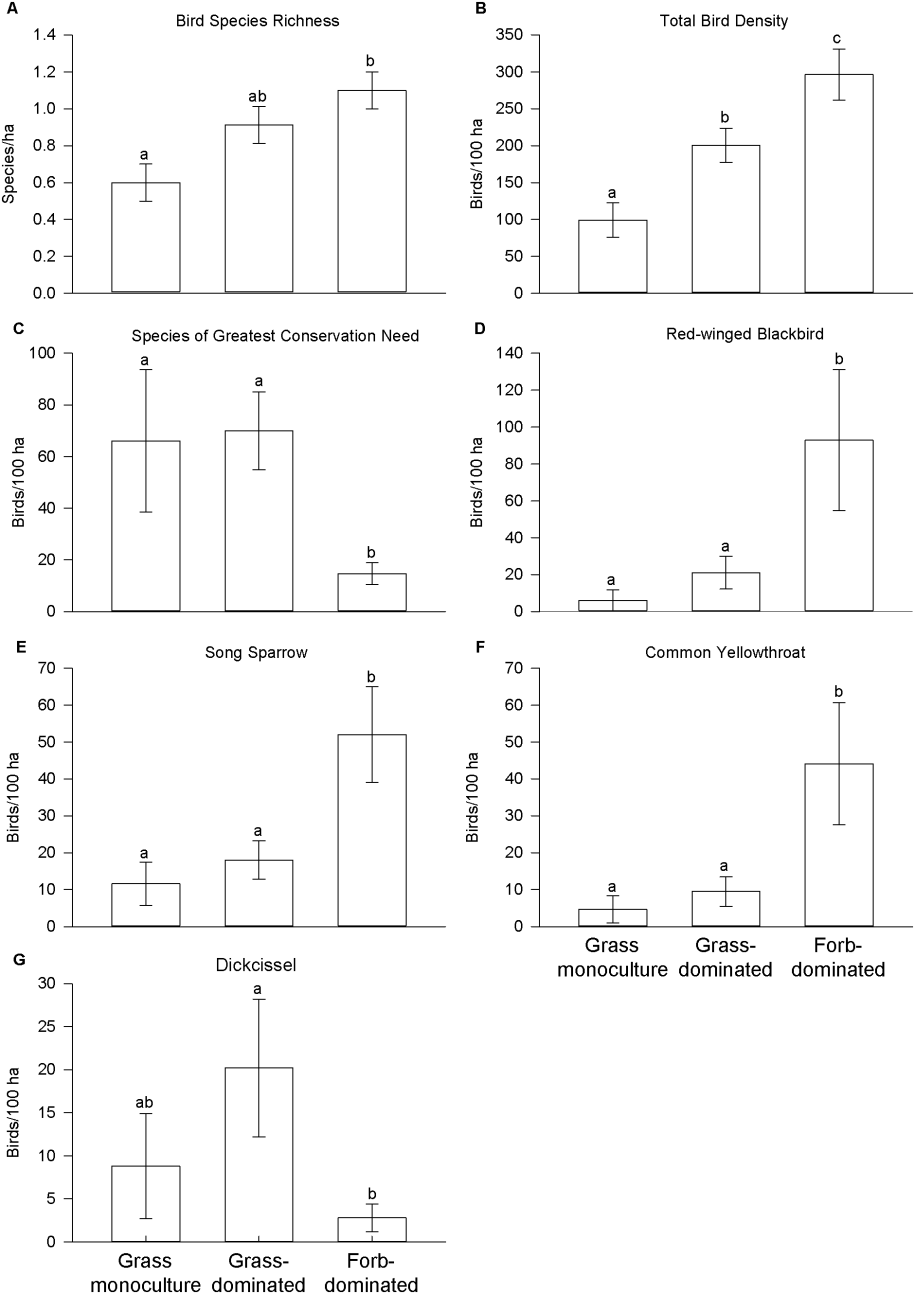
Predicted means of bird metrics in the three grassland field types. Error bars are ±1 SE. Bars with the same letter above them are not significantly different.

Among grassland fields, total bird density was greatest in forb-dominated fields, lower in grass-dominated fields, and lowest in grass monocultures (Fig. 2). Species richness was greater in forb-dominated fields than grass monocultures, and the densities of Red-winged Blackbirds, Song Sparrows, and Common Yellowthroats were greater in forb-dominated fields than grass monocultures and grass-dominated fields. However, SGCN density was lowest in forb-dominated fields and Dickcissel (also a SGCN) density was lower in forb-dominated fields than in grass-dominated fields.

Both local vegetation and landscape variables were related to bird metrics, as indicated by the most supported models (Table S4) and model-averaged parameter estimates (Table S5). Forb cover was consistently positively related to bird species richness and the density of total birds and Red-winged Blackbirds. Warm-season grass cover was negatively related to Red-winged Blackbird and Common Yellowthroat densities. SGCN density was negatively associated vertical vegetation density. The square of vertical vegetation density was negatively associated with the density of total birds, Red-winged Blackbirds, Song Sparrows, and Common Yellowthroats, indicating unimodal relationships with these bird metrics and vegetation density. The amount of grassland habitat within 1 km was positively related to bird species richness and the density of total birds, SGCNs, Red-winged Blackbirds, Common Yellowthroats, and Dickcissels.

**Figure 3:**
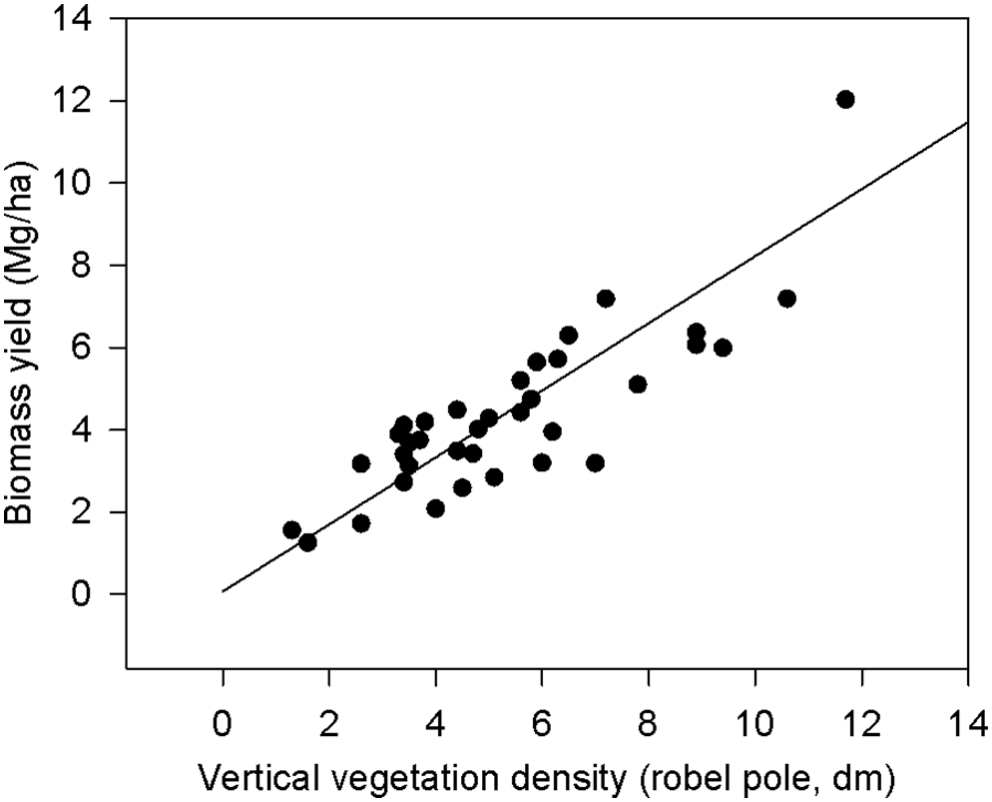
Relationship between biomass yield and vertical vegetation density in grassland fields. Predicted (solid line) and observed values (closed circles) are shown.

Fall biomass yields on grassland fields ranged from 1.24 to 12.02 Mg/ha. Biomass yield (mean ± SE) in grass monocultures (6.4±1.03 Mg/ha) was greater than grass-dominated (3.58±0.85 Mg/ha, *t* = 3.28, *P* = 0.003) and forb-dominated (4.67±0.86 Mg/ha, *t* = 2.02, *P* = 0.05) fields. Yields were similar between grass-dominated and forb-dominated fields (*t* = 1.7, *P* = 0.10). Fall biomass yield was positively related to summer canopy cover (*t* = 7.3, *P*<0.001) and vertical density (*t* = 14.12, *P*<0.001; Fig. 3), but was not related to plant species richness (*t* = 0.83, *P* = 0.44) or other summer vegetation characteristics. Among the six grassland fields for which we estimated biomass yields in both 2011 and 2012, yields were greater in 2011 (7.22±1.20 Mg/ha) than in 2012 (4.14±0.95 Mg/ha), suggesting a large yearly change in biomass yield, most likely due to the severe drought experienced in Wisconsin in the summer of 2012 [35].

**Figure 4:**
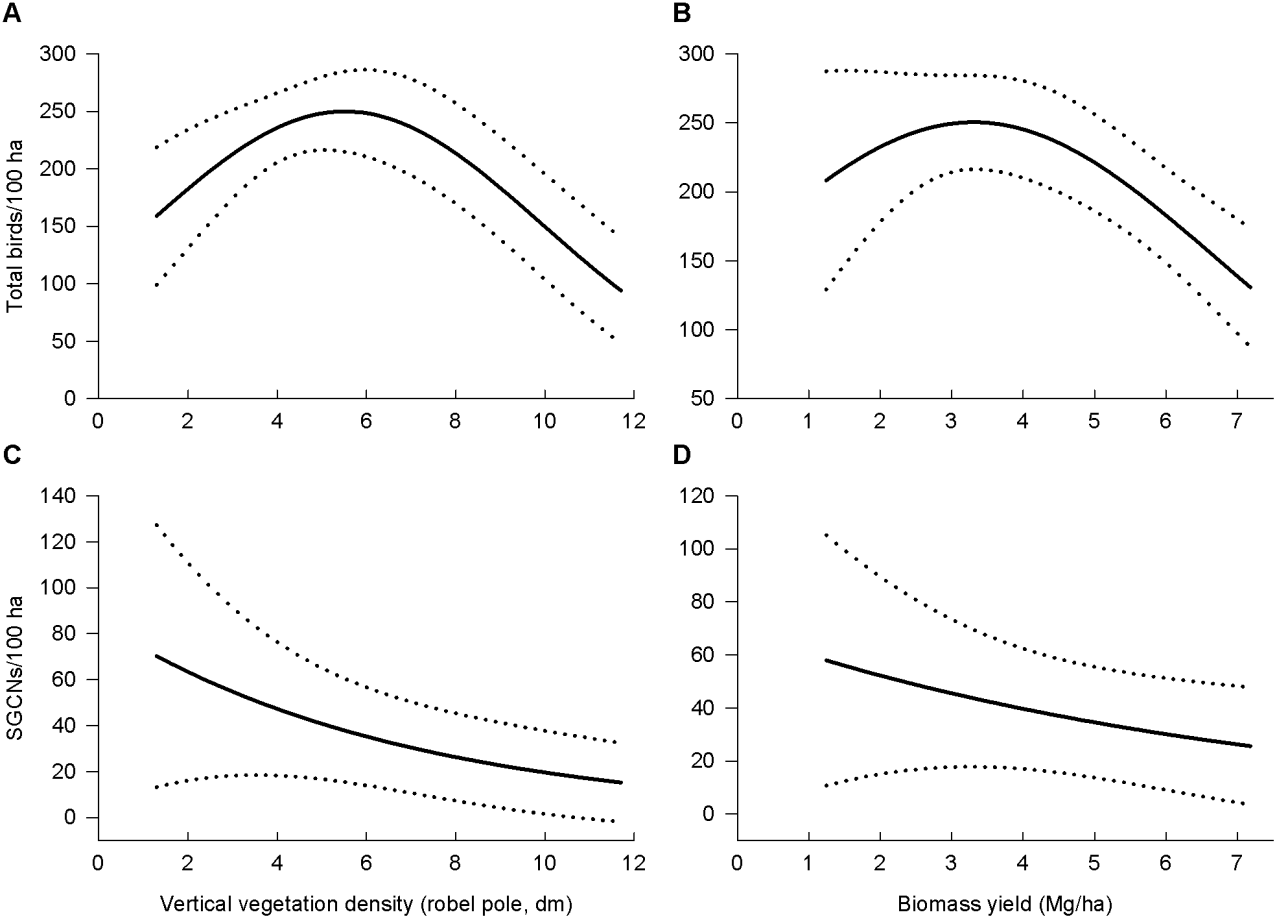
Predicted relationships of bird densities with vertical vegetation density and biomass yields. Total bird density (a–b) and SGCN density (c–d) are shown. Dotted lines are 95% confidence intervals.

Predicted total bird density had unimodal relationships with vertical vegetation density and biomass yield and declined above approximately 4 Mg biomass/ha (Fig. 4). We found similar unimodal trends for Red-winged Blackbird and Common Yellowthroat densities. Predicted SGCN density was negatively related to both vertical vegetation density and biomass yield.

## Discussion

Perennial, warm-season grasslands representing potential bioenergy crops provided habitat for a variety of grassland birds, including 10 SGCNs in Wisconsin. Bird densities for most species were greater in perennial grassland field types than cornfields. Recent increases in commodity prices have created incentives for farm owners to convert grasslands into corn [5], [47]. Our results concur with previous studies that suggest that an increase in corn production to meet bioenergy demand would be detrimental to grassland bird populations [4], [10], [15], [17]. Bird species of conservation concern may experience greater negative impacts of increased corn production than more common species [4]. In general, further conversion of grasslands to annual row crops would decrease wildlife habitat, increase carbon emissions, and harm water quality [10].

Among the field types we studied, most bird metrics were greater in forb-dominated fields but were similar between grass monocultures and grass-dominated fields. Forb cover was also positively associated with bird species richness and total bird density. Because forb cover was positively correlated with plant species richness, we infer that most birds were attracted to sites with greater plant species richness and a higher ratio of forbs to grasses. These results support research suggesting perennial bioenergy grasslands with greater plant diversity and forb cover would provide better bird habitat than less diverse crops [10], [17], [40]. Diverse grasslands provide food for herbivores and nectivores, which may increase insect diversity and in turn provide additional food resources for birds [10], [40], [58]. Diverse grasslands could also increase populations of insect crop-pollinators and pest natural enemies [7], [10], [59]. However, feedstocks with lower grass cover and greater plant diversity may have lower bioenergy conversion efficiencies and therefore may not be as profitable as less diverse feedstocks [29], [30], [31]. Additionally, grassland fields that start out with high plant diversity may decrease in diversity over time due to repeated annual harvests or dominance by some grass species [29].

In our study, predicted SGCN and Dickcissel densities were lowest in forb-dominated fields compared to less diverse grasslands. These trends in SGCN and Dickcissel densities were likely driven by the habitat structure of the fields. Most grassland SGCNs in Wisconsin prefer grasslands with low to moderate vertical density and are more responsive to habitat structure than to plant species composition [22], [60]. Average vertical density was greater in forb-dominated fields than in grass monocultures or grass-dominated fields. Thus, the forb-dominated fields were likely too tall and dense for most SGCNs. SGCN density in grass monocultures may have been inflated because one grass monoculture field had been colonized by a few common milkweed (*Asclepias syriaca*) plants that were popular perches for Dickcissels, and another grass monoculture field was adjacent to a florally diverse field that appeared to attract SGCNs to the area. Further, grass-dominated fields contained an average of 11% forbs, which can be enough to accommodate the needs of many SGCNs [60].

Habitat amount generally has stronger effects on biodiversity than habitat configuration [61]. The amount of grassland habitat in the landscapes surrounding our study sites was positively associated with most bird metrics, suggesting grassland bioenergy fields would be more beneficial for grassland birds if they are near other grassland parcels [10], [17], [25], [62]. We did not find strong evidence of a relationship between most bird metrics and the amount of agriculture in the landscape. Agriculture and forest cover were negatively correlated, suggesting that the bird communities in grasslands were not strongly influenced by forest cover in the landscape. This contrasts with other studies that have found that grassland birds are negatively influenced by the proportion of forest in the landscape [15], [20], [40], [63], [64], and may be explained by the fairly low average amount of forest cover (17%) in the agriculture-dominated landscape of our study area.

Based on estimates of potential grassland biomass yields from research in the upper Midwest, the biomass yields from the fields in our study represent plausible, future, commercial-scale, grassland biomass yields [13], [42]. Our results agree with others who suggest that grass monocultures would produce greater biomass yields than grass-forb mixtures [29], [32], [41], [65]. Notably, biomass yield across all of our grassland sites was not related to plant species richness, contrasting with other studies that have suggested such a relationship [e.g.,12,30]. Thus, we found that although grass monocultures had the greatest biomass yields, increasing plant diversity in general in perennial grassland fields may have little effect on biomass yields. The frequency, timing, and amount of grassland biomass harvests will also impact biomass yields and bird habitat quality and should be considered when developing harvesting strategies [2], [10], [21], [29], [66].

Our analyses relating breeding bird metrics to biomass yields focuses attention on the possible tradeoffs between maintaining bird habitat and producing grassland biomass. Our results support previous suggestions that bioenergy production fields managed for the greatest biomass yield would be of lesser value to bird communities than lower-yielding fields [5], [10], [40]. Our analyses relating bird metrics to biomass yields may have been influenced by several factors. For example, our biomass yield estimates were obtained following a record-setting drought in 2012, so yields in more average growing seasons could be substantially greater and may influence bird response. Additionally, total bird density was dominated by the most common species (e.g., Red-winged Blackbirds and Common Yellowthroats), and SGCN density was dominated by Dickcissels, therefore trends for those metrics may be driven by a few species. We note that our analyses relate breeding bird densities measured in summer to biomass yields measured in October; this temporal distinction is important because grassland biomass will be harvested well after the breeding season.

Of particular importance for conservation is the impact that grassland biomass fields could have on SGCN populations. We found that SGCN density was negatively associated with vertical vegetation density and biomass yields, suggesting that tall and dense grassland bioenergy fields managed for the greatest biomass yields would be of lesser value to imperiled grassland bird species. If future perennial grass production is designed to maximize biomass yields by creating monocultures of tall and dense grasses, although some habitat generalists (e.g., Song Sparrow) or species that tolerate dense grasses (e.g., Sedge Wren [*Cistothorus platensis*]) may adapt [17], [67], biodiversity will generally be reduced [7]. We agree with Webster et al. [7∶457] who suggest that extracting “optimal rather than maximal quantities of biomass” may be necessary to maintain or enhance biodiversity.

In this study we used species richness and bird densities to test for habitat preferences among birds that utilize grassland habitats. We acknowledge that greater richness and densities of birds does not necessarily indicate higher quality habitat for birds and that reproductive success may differ among grassland biomass crops [68], [69]. Reproductive success in grassland biomass fields may vary with differences in predation, brood parasitism, food availability, and other factors. These factors will depend on both the local habitat characteristics and the landscape context around the crops. Management intensity and the timing of biomass harvests could also affect reproductive success, and nest losses could be minimized if sustainable harvesting practices were followed [33].

There is a growing understanding that working agricultural lands will need to produce commodities as well as provide environmental benefits [8], [70], [71]. Although recent profitability analyses indicate that cellulosic bioenergy crops such as switchgrass and mixed grasses would be less profitable than corn as a biofuel feedstock [72], these studies do not include the costs of biodiversity loss, soil loss, and other societal and ecological impacts associated with conventional, annual row crop agriculture. With appropriate financial incentives, farm owners could be motivated to use diverse grassland bioenergy plantings, and sacrifice some biomass yield, in order to support greater agricultural multifunctionality and meet conservation goals [8], [10], [17].

## Supporting Information

Table S1 Field types, common plant species, and estimated biomass yields of grassland study sites in southern Wisconsin.
(DOCX)

Table S2 Soil characteristics, determined from the SSURGO Database [73], of grassland study sites in southern Wisconsin.
(DOCX)

Table S3 Mean bird densities (birds/100 ha) in cornfields and the three grassland field types, and the total number of sites where each species was detected, in 2011–2012.
(DOCX)

Table S4 Top models with Δ AIC_c_≤2.0 for each of the seven bird metrics. Predictor variables in the full (global) models of each response variable included % cover of Forbs (Forbs) and warm-season grasses (WSG), vertical vegetation density (Robel), Robel^2^, and % agriculture (Ag) and grassland (Grassland) within 1 km.
(DOCX)

Table S5 Model-averaged parameter estimates and 95% unconditional confidence limits of explanatory variables included in models of the seven bird metrics. Predictor variables in the full (global) models of each response variable included % cover of Forbs (Forbs) and warm-season grasses (WSG), vertical vegetation density (Robel), Robel^2^, and % agriculture (Ag) and grassland (Grassland) within 1 km. Confidence intervals that do not overlap zero are bolded.
(DOCX)

Dataset S1 **Abundance of individual bird species during point counts at each study site.** See Metadata worksheet for definition of variables.
(XLSX)

Dataset S2 **Vegetation characteristics, biomass yields, and landscape attributes of grassland study sites.** See Metadata worksheet for definition of variables.
(XLSX)
