# Supplementary material for: Bird Communities and Biomass Yields in Potential Bioenergy Grasslands
Source: PLoS One. 2014 Oct 9;9(10):e109989. doi: 10.1371/journal.pone.0109989 (PMC4192549; doi:10.1371/journal.pone.0109989)
Supplement: Table S1 — Field types, common plant species, and estimated biomass yields of grassland study sites in southern Wisconsin. (DOCX) [file pone.0109989.s001.docx]

**Table S1:** Field types, common plant species, and estimated biomass yields of grassland study sites in southwestern Wisconsin.

|  |  | **Common grass and forb species** | |  |
| --- | --- | --- | --- | --- |
| **Field #** | **Field type** | **Common name** | **Scientific name^a^** | **Biomass yield (Mg/ha)^b^** |
| 1 | Grass monoculture | Big bluestem | *Andropogon gerardii* | 6.29 |
| 2 | Grass monoculture | Big bluestem | *Andropogon gerardii* | 4.19 |
| 3 | Grass monoculture | Indiangrass | *Sorghastrum nutans* | 3.68 |
| 4 | Grass monoculture | Switchgrass | *Panicum virgatum* | 9.60 |
| 5 | Grass monoculture | Switchgrass | *Panicum virgatum* | 3.74 |
|  |  | Indiangrass | *Sorghastrum nutans* |  |
| 6 | Grass-dominated | Switchgrass | *Panicum virgatum* | 1.55 |
|  |  | Kentucky bluegrass | *Poa pratensis* |  |
|  |  | Reed canarygrass | *Phalaroides arundinacea* |  |
|  |  | Canada goldenrod | *Solidago canadensis* |  |
|  |  | Red clover | *Trifolium pratense* |  |
|  |  | Alsike clover | *Trifolium hybridum* |  |
|  |  | Queen anne's lace | *Daucus carota* |  |
| 7 | Grass-dominated | Indiangrass | *Sorghastrum nutans* | 5.64 |
|  |  | Big bluestem | *Andropogon gerardii* |  |
|  |  | Canada goldenrod | *Solidago canadensis* |  |
|  |  | Stiff goldenrod | *Solidago rigida* |  |
|  |  | Wild bergamot | *Monarda fistulosa* |  |
| 8 | Grass-dominated | Big bluestem | *Andropogon gerardii* | 5.19 |
|  |  | Reed canarygrass | *Phalaroides arundinacea* |  |
|  |  | Canada goldenrod | *Solidago canadensis* |  |
| 9 | Grass-dominated | Big bluestem | *Andropogon gerardii* | 3.12 |
|  |  | Kentucky bluegrass | *Poa pratensis* |  |
|  |  | Common milkweed | *Asclepias syriaca* |  |
| 10 | Grass-dominated | Indiangrass | *Sorghastrum nutans* | 4.01 |
|  |  | Eastern daisy fleabane | *Erigeron annuus* |  |
|  |  | Canada thistle | *Cirsium arvense* |  |
| 11 | Grass-dominated | Big bluestem | *Andropogon gerardii* | 3.47 |
|  |  | Little bluestem | *Schizachyrium scoparium* |  |
|  |  | Pinnate prairie coneflower | *Ratibida pinnata* |  |
|  |  | Canada goldenrod | *Solidago canadensis* |  |
|  |  | Golden alexanders | *Zizia aurea* |  |
|  |  | Eastern daisy fleabane | *Erigeron annuus* |  |
|  |  | Wild bergamot | *Monarda fistulosa* |  |
| 12 | Grass-dominated | Big bluestem | *Andropogon gerardii* | 3.39 |
|  |  | Little bluestem | *Schizachyrium scoparium* |  |
|  |  | Switchgrass | *Panicum virgatum* |  |
|  |  | Showy ticktrefoil | *Desmodium canadense* |  |
|  |  | Wild bergamot | *Monarda fistulosa* |  |
|  |  | Pinnate prairie coneflower | *Ratibida pinnata* |  |
|  |  | Blackeyed Susan | *Rudbeckia hirta* |  |
| 13 | Grass-dominated | Little bluestem | *Schizachyrium scoparium* | 1.71 |
|  |  | Indiangrass | *Sorghastrum nutans* |  |
|  |  | Kentucky bluegrass | *Poa pratensis* |  |
|  |  | Smooth oxeye | *Heliopsis helianthoides* |  |
|  |  | Roundhead lespedeza | *Lespedeza capitata* |  |
|  |  | Whorled milkweed | *Asclepias variegata* |  |
|  |  | Annual ragweed | *Ambrosia artemesiifolia* |  |
| 14 | Grass-dominated | Big bluestem | *Andropogon gerardii* | 4.28 |
|  |  | Indiangrass | *Sorghastrum nutans* |  |
|  |  | Switchgrass | *Panicum virgatum* |  |
|  |  | Wild bergamot | *Monarda fistulosa* |  |
|  |  | Red clover | *Trifolium pratense* |  |
|  |  | Flowering spurge | *Euphorbia corollata* |  |
| 15 | Grass-dominated | Switchgrass | *Panicum virgatum* | 4.11 |
|  |  | Big bluestem | *Andropogon gerardii* |  |
|  |  | Wild parsnip | *Pastinaca sativa* |  |
|  |  | White sweet-clover | *Melilotus alba* |  |
|  |  | Yellow foxtail | *Setaria glauca* |  |
|  |  | Whorled milkweed | *Asclepias variegata* |  |
| 16 | Grass-dominated | Indiangrass | *Sorghastrum nutans* | 2.72 |
|  |  | Switchgrass | *Panicum virgatum* |  |
|  |  | Kentucky bluegrass | *Poa pratensis* |  |
|  |  | Canada goldenrod | *Solidago canadensis* |  |
|  |  | Alsike clover | *Trifolium hybridum* |  |
|  |  | Narrowleaf hawkweed | *Hieracium umbellatum* |  |
| 17 | Grass-dominated | Big bluestem | *Andropogon gerardii* | 3.16 |
|  |  | Little bluestem | *Schizachyrium scoparium* |  |
|  |  | Kentucky bluegrass | *Poa pratensis* |  |
|  |  | Red clover | *Trifolium pratense* |  |
|  |  | Wild bergamot | *Monarda fistulosa* |  |
| 18 | Grass-dominated | Big bluestem | *Andropogon gerardii* | 1.24 |
|  |  | Little bluestem | *Schizachyrium scoparium* |  |
|  |  | Indiangrass | *Sorghastrum nutans* |  |
|  |  | Canada goldenrod | *Solidago canadensis* |  |
|  |  | Common dandelion | *Taraxacum officinale* |  |
|  |  | Wild bergamot | *Monarda fistulosa* |  |
|  |  | Purple prairie clover | *Dalea purpurea* |  |
| 19 | Grass-dominated | Indiangrass | *Sorghastrum nutans* | 3.90 |
|  |  | Little bluestem | *Schizachyrium scoparium* |  |
|  |  | Big bluestem | *Andropogon gerardii* |  |
|  |  | Kentucky bluegrass | *Poa pratensis* |  |
|  |  | Reed canarygrass | *Phalaroides arundinacea* |  |
|  |  | Smooth brome | *Bromus inermis* |  |
|  |  | Queen anne's lace | *Daucus carota* |  |
|  |  | Wild parsnip | *Pastinaca sativa* |  |
|  |  | Common dandelion | *Taraxacum officinale* |  |
|  |  | Pinnate prairie coneflower | *Ratibida pinnata* |  |
|  |  | Wild bergamot | *Monarda fistulosa* |  |
|  |  | Flowering spurge | *Euphorbia corollata* |  |
| 20 | Forb-dominated | Big bluestem | *Andropogon gerardii* | 4.58 |
|  |  | Virginia mountain mint | *Pycnanthemum virginianum* |  |
|  |  | Wholeleaf rosinweed | *Silphium integrifolium* |  |
|  |  | Compassplant | *Silphium laciniatum* |  |
| 21 | Forb-dominated | Switchgrass | *Panicum virgatum* | 2.83 |
|  |  | Big bluestem | *Andropogon gerardii* |  |
|  |  | Compassplant | *Silphium laciniatum* |  |
|  |  | Stiff goldenrod | *Solidago rigida* |  |
|  |  | Golden alexanders | *Zizia aurea* |  |
|  |  | White wild indigo | *Baptesia alba* |  |
| 22 | Forb-dominated | Indiangrass | *Sorghastrum nutans* | 3.42 |
|  |  | Little bluestem | *Schizachyrium scoparium* |  |
|  |  | Big bluestem | *Andropogon gerardii* |  |
|  |  | Common dandelion | *Taraxacum officinale* |  |
|  |  | Golden alexanders | *Zizia aurea* |  |
|  |  | Stiff goldenrod | *Solidago rigida* |  |
|  |  | Field thistle | *Cirsium discolor* |  |
| 23 | Forb-dominated | Big bluestem | *Andropogon gerardii* | 3.95 |
|  |  | Common milkweed | *Asclepias syriaca* |  |
|  |  | Wild bergamot | *Monarda fistulosa* |  |
|  |  | Canada goldenrod | *Solidago canadensis* |  |
|  |  | Cup plant | *Silphium perfoliatum* |  |
|  |  | New England aster | *Symphyotrichium novae-angliae* |  |
| 24 | Forb-dominated | Indiangrass | *Sorghastrum nutans* | 3.19 |
|  |  | Little bluestem | *Schizachyrium scoparium* |  |
|  |  | Wild bergamot | *Monarda fistulosa* |  |
|  |  | Blackeyed Susan | *Rudbeckia hirta* |  |
|  |  | Canada goldenrod | *Solidago canadensis* |  |
|  |  | Aster spp. | *Symphyotrichum spp.* |  |
|  |  | Spiny plumeless thistle | *Carduus acanthoides* |  |
| 25 | Forb-dominated | Big bluestem | *Andropogon gerardii* | 5.23 |
|  |  | Stiff goldenrod | *Solidago rigida* |  |
|  |  | Canada goldenrod | *Solidago canadensis* |  |
|  |  | Cup plant | *Silphium perfoliatum* |  |
|  |  | Prairie rosinweed | *Silphium terebinthinaceum* |  |
|  |  | Wholeleaf rosinweed | *Silphium integrifolium* |  |
|  |  | Wild bergamot | *Monarda fistulosa* |  |
| 26 | Forb-dominated | Big bluestem | *Andropogon gerardii* | 4.32 |
|  |  | Indiangrass | *Sorghastrum nutans* |  |
|  |  | Kentucky bluegrass | *Poa pratensis* |  |
|  |  | Canada goldenrod | *Solidago canadensis* |  |
|  |  | Wild bergamot | *Monarda fistulosa* |  |
|  |  | Pinnate prairie coneflower | *Ratibida pinnata* |  |
|  |  | Golden alexanders | *Zizia aurea* |  |
|  |  | Common dandelion | *Taraxacum officinale* |  |
| 27 | Forb-dominated | Indiangrass | *Sorghastrum nutans* | 4.42 |
|  |  | Switchgrass | *Panicum virgatum* |  |
|  |  | Wild bergamot | *Monarda fistulosa* |  |
|  |  | Wild lettuce | *Lactuca canadensis* |  |
|  |  | Canada goldenrod | *Solidago canadensis* |  |
|  |  | Illinois Tick-trefoil | *Desmodium illinoense* |  |
| 28 | Forb-dominated | Big bluestem | *Andropogon gerardii* | 4.48 |
|  |  | Canada goldenrod | *Solidago canadensis* |  |
|  |  | Wild bergamot | *Monarda fistulosa* |  |
|  |  | Canada thistle | *Cirsium arvense* |  |
|  |  | New England aster | *Symphyotrichium novae-angliae* |  |
| 29 | Forb-dominated | Switchgrass | *Panicum virgatum* | 4.22 |
|  |  | Indiangrass | *Sorghastrum nutans* |  |
|  |  | Canada goldenrod | *Solidago canadensis* |  |
|  |  | Red elderberry | *Sambucus racemosa* |  |
|  |  | Narrowleaf hawkweed | *Hieracium umbellatum* |  |
| 30 | Forb-dominated | Switchgrass | *Panicum virgatum* | 6.14 |
|  |  | Canada goldenrod | *Solidago canadensis* |  |
|  |  | Spiny plumeless thistle | *Carduus acanthoides* |  |
|  |  | Wild parsnip | *Pastinaca sativa* |  |
|  |  | Crown vetch | *Coronilla varia* |  |

^a^ Commonly encountered grass and forb species at each site are listed with common grasses first followed by common forbs.

^b^ For sites surveyed in two years, the average estimated biomass yield across years is reported.
