# Supplementary material for: Bird Communities and Biomass Yields in Potential Bioenergy Grasslands
Source: PLoS One. 2014 Oct 9;9(10):e109989. doi: 10.1371/journal.pone.0109989 (PMC4192549; doi:10.1371/journal.pone.0109989)
Supplement: Table S2 — Soil characteristics, determined from the SSURGO Database [73], of grassland study sites in southern Wisconsin. (DOCX) [file pone.0109989.s002.docx]

**Table S2:** Soil characteristics of grassland study sites in southern Wisconsin.

| **Field #** | **Soil Type** |
| --- | --- |
| 1 | Grellton fine sandy loam, 1 to 6 percent slopes |
| 2 | Dodgeville silt loam, deep, 2 to 6 percent slopes, moderately eroded |
| 3 | Sparta loamy sand, 1 to 6 percent slopes |
| 4 | Jackson silt loam, 2 to 6 percent slopes |
| 5 | Billett sandy loam, 1 to 6 percent slopes |
| 6 | Dubuque silt loam, 2 to 6 percent slopes, moderately eroded |
| 7 | McHenry silt loam, 6 to 12 percent slopes, eroded |
| 8 | Batavia silt loam, gravelly substratum, 0 to 2 percent slopes |
| 9 | Plano silt loam, 2 to 6 percent slopes |
| 10 | Kegonsa silt loam, 2 to 6 percent slopes |
| 11 | Lapeer fine sandy loam, 2 to 6 percent slopes |
| 12 | Lapeer fine sandy loam, 2 to 6 percent slopes |
| 13 | Sparta loamy sand, 1 to 6 percent slopes |
| 14 | Plano silt loam, 2 to 6 percent slopes |
| 15 | Ashdale silt loam, 6 to 12 percent slopes, moderately eroded |
| 16 | Orion silt loam |
| 17 | Batavia silt loam, gravelly substratum, 6 to 12 percent slopes, eroded |
| 18 | Plano silt loam, 2 to 6 percent slopes |
| 19 | Kidder silt loam, 2 to 6 percent slopes, eroded |
| 20 | Virgil silt loam, gravelly substratum, 0 to 3 percent slopes |
| 21 | Wauconda silt loam, 2 to 6 percent slopes |
| 22 | Ringwood silt loam, 2 to 6 percent slopes |
| 23 | Dresden silt loam, 2 to 6 percent slopes |
| 24 | Sebewa silt loam, clayey substratum |
| 25 | Watseka loamy fine sand |
| 26 | Plano silt loam, 6 to 12 percent slopes, eroded |
| 27 | Ripon silt loam, 6 to 12 percent slopes, eroded |
| 28 | Troxel silt loam, 1 to 3 percent slopes |
| 29 | Batavia silt loam, gravelly substratum, 2 to 6 percent slopes |
| 30 | Rotamer loam, 2 to 6 percent slopes, eroded |
