# Supplementary material for: Bird Communities and Biomass Yields in Potential Bioenergy Grasslands
Source: PLoS One. 2014 Oct 9;9(10):e109989. doi: 10.1371/journal.pone.0109989 (PMC4192549; doi:10.1371/journal.pone.0109989)
Supplement: Table S3 — Mean bird densities (birds/100 ha) in cornfields and the three grassland field types, and the total number of sites where each species was detected, in 2011–2012. (DOCX) [file pone.0109989.s003.docx]

**Table S3:** Mean bird densities (birds/100 ha) in cornfields and the three grassland field types, and the total number of sites where each species was detected, in 2011-2012.^a,b^

|  | **Corn** | | **Grass monoculture** | | **Grass-dominated** | | **Forb-dominated** | |  |
| --- | --- | --- | --- | --- | --- | --- | --- | --- | --- |
| **Species name** | **Mean** | **SE** | **Mean** | **SE** | **Mean** | **SE** | **Mean** | **SE** | **Number of sites detected** |
| Song Sparrow^c^ (*Melospiza melodia*) | 1 | 1 | 21.2 | 11.1 | 27.3 | 10.1 | 53.5 | 6.3 | 24 |
| Red-winged Blackbird^c^ (*Agelaius phoeniceus*) | 0 | 0 | 8.5 | 4 | 52.3 | 18.2 | 166.9 | 34 | 20 |
| Common Yellowthroat^c^ (*Geothlypis trichas*) | 0 | 0 | 8.5 | 5.2 | 21.2 | 9.6 | 58.8 | 9.6 | 19 |
| Dickcissel^d, f^ (*Spiza Americana*) | 0 | 0 | 24.4 | 8.8 | 34.9 | 11.3 | 9.2 | 5.3 | 16 |
| Grasshopper Sparrow^d,f^ (*Ammodramus savannarum*) | 0 | 0 | 19.1 | 7.8 | 15.9 | 7.2 | 2.4 | 2.4 | 10 |
| Henslow's Sparrow^d,f^ (*Ammodramus henslowii*) | 0 | 0 | 0 | 0 | 34.1 | 12.8 | 0 | 0 | 7 |
| American Goldfinch^c^ (*Spinus tristis*) | 1 | 1 | 0 | 0 | 3.8 | 2.6 | 13.5 | 8.8 | 6 |
| Eastern Meadowlark^d,f^ (*Sturnella magna*) | 0 | 0 | 5.3 | 4.1 | 5.3 | 4 | 2.4 | 1.9 | 6 |
| Bobolink^d,f^ (*Dolichonyx oryzivorus*) | 0 | 0 | 0 | 0 | 12.1 | 8.4 | 0 | 0 | 4 |
| Horned Lark^d^ (*Eremophila alpestris*) | 5.8 | 3.3 | 1.1 | 1.1 | 0 | 0 | 0 | 0 | 4 |
| Clay-colored Sparrow ^e^ (*Spizella pallida*) | 0 | 0 | 0 | 0 | 0.8 | 0.8 | 10.6 | 5.9 | 4 |
| Field Sparrow^e,f^ (*Spizella pusilla*) | 0 | 0 | 0 | 0 | 2.3 | 1.6 | 1.4 | 1 | 4 |
| American Robin^c^ (*Turdus migratorius*) | 0 | 0 | 1.1 | 1.1 | 0.8 | 0.8 | 1 | 1 | 3 |
| Brown-headed Cowbird^e^ (*Molothrus ater*) | 2.9 | 2.9 | 0 | 0 | 0 | 0 | 3.4 | 2.9 | 3 |
| Savannah Sparrow^d^ (*Passerculus sandwichensis*) | 1 | 1 | 0 | 0 | 2.3 | 1.6 | 0 | 0 | 3 |
| Sedge Wren^d^ (*Cistothorus platensis*) | 0 | 0 | 0 | 0 | 2.3 | 1.6 | 1.9 | 1.9 | 3 |
| Northern Harrier^d,f^ (*Circus cyaneus*) | 0 | 0 | 0 | 0 | 1.5 | 1.5 | 1.9 | 1.9 | 2 |
| Ring-necked Pheasant ^e^ (*Phasianus colchicus*) | 0 | 0 | 0 | 0 | 0 | 0 | 1.9 | 1.3 | 2 |
| Gray Catbird^c^ (*Dumetella carolinensis*) | 0 | 0 | 0 | 0 | 0 | 0 | 1 | 1 | 1 |
| Ruby-throated Hummingbird^c^ (*Archilochus colubris*) | 0 | 0 | 0 | 0 | 0.8 | 0.8 | 0 | 0 | 1 |
| Upland Sandpiper^d,f^ (*Bartramia longicauda*) | 0 | 0 | 0 | 0 | 0 | 0 | 0.5 | 0.5 | 1 |
| Willow Flycatcher^c,f^ (*Empidonax traillii*) | 0 | 0 | 0 | 0 | 0 | 0 | 2.4 | 2.4 | 1 |
| Chipping Sparrow^c^ (*Spizella passerina*) | 0 | 0 | 0 | 0 | 0 | 0 | 1 | 1 | 1 |
| Vesper Sparrow^d,f^ (*Pooecetes gramineus*) | 0 | 0 | 0 | 0 | 3.8 | 3.8 | 0 | 0 | 1 |
| Common Grackle^c^ (*Quiscalus quiscula*) | 0 | 0 | 0 | 0 | 0.8 | 0.8 | 0 | 0 | 1 |
| House Sparrow^c^ (*Passer domesticus*) | 6.8 | 6.8 | 0 | 0 | 0 | 0 | 0 | 0 | 1 |
| Swamp Sparrow (*Melospiza georgiana*) | 0 | 0 | 0 | 0 | 0 | 0 | 1 | 1 | 1 |
| Orchard Oriole^c^ (*Icterus spurius*) | 0 | 0 | 0 | 0 | 0.8 | 0.8 | 0 | 0 | 1 |
| Indigo Bunting^c^ (*Passerina cyanea*) | 0 | 0 | 0 | 0 | 0 | 0 | 1 | 1 | 1 |

^a^ Sample sizes: cornfields (*n* = 11), grass monoculture (*n* = 5), grass-dominated (*n* = 14), forb-dominated (*n* = 11).

^b^ Means were calculated by first averaging the surveys within one year and then averaging among years.

^c-e^ Breeding habitat preference for each species [[based on 55](#_ENREF_55)]: ^c^ Habitat generalist that commonly occurs in grasslands; ^d^ Grassland obligate; ^e^ Requires grasslands during their breeding cycle.

^f^ Species of greatest conservation need in Wisconsin.
