# Supplementary material for: Bird Communities and Biomass Yields in Potential Bioenergy Grasslands
Source: PLoS One. 2014 Oct 9;9(10):e109989. doi: 10.1371/journal.pone.0109989 (PMC4192549; doi:10.1371/journal.pone.0109989)
Supplement: Table S4 — Top models with Δ AICc≤2.0 for each of the seven bird metrics. Predictor variables in the full (global) models of each response variable included % cover of Forbs (Forbs) and warm-season grasses (WSG), vertical vegetation density (Robel), Robel2, and % agriculture (Ag) and grassland (Grassland) within 1 km. (DOCX) [file pone.0109989.s004.docx]

| **Table S4:** Top models of bird metrics with Δ AIC_c_ ≤ 2.0. Predictor variables in the full (global) models of each response variable included % cover of Forbs (Forbs) and warm-season grasses (WSG), vertical vegetation density (Robel), Robel^2^, and % agriculture (Ag) and grassland (Grassland) within 1 km. | | | | | | |
| --- | --- | --- | --- | --- | --- | --- |
|  | **Predictor variables in model^a^** | |  |  |  |  |
| **Response variable** | **Local vegetation variables** | **Landscape variables** | **K** | **AIC_c_** | **Δ AIC_c_** | **w_i_** |
| Bird species richness | + Forbs | + Grassland | 5 | 382.35 | 0.00 | 0.22 |
|  | + Forbs |  | 4 | 384.26 | 1.91 | 0.08 |
|  | + Forbs − Robel | + Grassland | 6 | 384.29 | 1.94 | 0.08 |
| Total bird density | + Forbs + Robel − Robel^2^ | + Grassland | 7 | 590.70 | 0.00 | 0.40 |
|  | + Forbs + Robel − Robel^2^ | − Ag + Grassland | 8 | 591.72 | 1.02 | 0.24 |
|  | + Forbs − WSG + Robel − Robel^2^ | + Grassland | 8 | 592.35 | 1.65 | 0.18 |
| SGCN | − Robel + Robel^2^ | + Grassland | 6 | 356.26 | 0.00 | 0.08 |
|  | + WSG − Robel | + Grassland | 6 | 356.48 | 0.22 | 0.07 |
|  | − Robel | + Grassland | 5 | 357.15 | 0.89 | 0.05 |
|  | − Forbs | − Ag + Grassland | 6 | 357.27 | 1.01 | 0.05 |
|  | + WSG − Robel | − Ag + Grassland | 7 | 357.32 | 1.06 | 0.04 |
|  | − Forbs − Robel | + Grassland | 6 | 357.45 | 1.19 | 0.04 |
|  | − Robel + Robel^2^ | − Ag + Grassland | 7 | 357.54 | 1.28 | 0.04 |
|  | − Forbs − Robel | − Ag + Grassland | 7 | 357.57 | 1.31 | 0.04 |
|  | − Forbs − Robel + Robel^2^ | + Grassland | 7 | 357.91 | 1.65 | 0.03 |
|  | − Robel |  | 4 | 358.09 | 1.83 | 0.03 |
|  | − Forbs − Robel + Robel^2^ | − Ag + Grassland | 8 | 358.14 | 1.88 | 0.03 |
|  | + WSG − Robel + Robel^2^ | + Grassland | 7 | 358.21 | 1.95 | 0.03 |
|  | − Forbs + WSG − Robel | − Ag + Grassland | 8 | 358.22 | 1.96 | 0.03 |
|  | − Forbs + WSG − Robel | + Grassland | 7 | 358.25 | 1.99 | 0.03 |
| Red-winged blackbird | + Forbs − WSG + Robel − Robel^2^ | + Grassland | 8 | 434.18 | 0.00 | 0.54 |
| Song sparrow | + Robel − Robel^2^ |  | 5 | 301.27 | 0.00 | 0.13 |
|  | − WSG + Robel − Robel^2^ |  | 6 | 301.74 | 0.47 | 0.10 |
|  | + Forbs + Robel − Robel^2^ |  | 6 | 302.14 | 0.87 | 0.08 |
|  | − WSG + Robel − Robel^2^ | + Ag | 7 | 303.02 | 1.75 | 0.05 |
|  | + Robel − Robel^2^ | + Ag | 6 | 303.13 | 1.85 | 0.05 |
|  | + Forbs + Robel − Robel^2^ | + Ag | 7 | 303.24 | 1.96 | 0.05 |
|  | + Robel − Robel^2^ | − Grassland | 6 | 303.27 | 2.00 | 0.05 |
| Common yellowthroat | − WSG + Robel − Robel^2^ | + Grassland | 7 | 277.66 | 0.00 | 0.30 |
|  | + Forbs − WSG + Robel − Robel^2^ | + Grassland | 8 | 278.86 | 1.20 | 0.17 |
|  | − WSG + Robel − Robel^2^ | − Ag + Grassland | 8 | 279.50 | 1.84 | 0.12 |
| Dickcissel | + WSG − Robel | + Ag + Grassland | 7 | 211.09 | 0.00 | 0.16 |
|  | + WSG − Robel | + Grassland | 6 | 211.77 | 0.68 | 0.11 |
|  | + WSG − Robel − Robel^2^ | + Ag + Grassland | 8 | 212.38 | 1.29 | 0.08 |
|  | − Forbs | + Grassland | 5 | 212.80 | 1.71 | 0.07 |
