# Supplementary material for: Bird Communities and Biomass Yields in Potential Bioenergy Grasslands
Source: PLoS One. 2014 Oct 9;9(10):e109989. doi: 10.1371/journal.pone.0109989 (PMC4192549; doi:10.1371/journal.pone.0109989)
Supplement: Table S5 — Model-averaged parameter estimates and 95% unconditional confidence limits of explanatory variables included in models of the seven bird metrics. Predictor variables in the full (global) models of each response variable included % cover of Forbs (Forbs) and warm-season grasses (WSG), vertical vegetation density (Robel), Robel2, and % agriculture (Ag) and grassland (Grassland) within 1 km. Confidence intervals that do not overlap zero are bolded. (DOCX) [file pone.0109989.s005.docx]

| **Table S5:** Model-averaged parameter estimates and 95% unconditional confidence limits of explanatory variables included in models of seven bird metrics. Predictor variables in the full (global) models of each response variable included % cover of Forbs (Forbs) and warm-season grasses (WSG), vertical vegetation density (Robel), Robel^2^, and % agriculture (Ag) and grassland (Grassland) within 1 km. Confidence intervals that do not overlap zero are bolded. | | | | |
| --- | --- | --- | --- | --- |
| **Response variable** | **Explanatory variable** | **LCL** | **Estimate** | **UCL** |
| Bird species richness | Forbs | **0.07** | **0.19** | **0.31** |
|  | WSG | −0.2 | −0.04 | 0.12 |
|  | Robel | −0.18 | −0.03 | 0.12 |
|  | Robel^2^ | −0.17 | −0.07 | 0.03 |
|  | Ag | −0.11 | 0.03 | 0.16 |
|  | Grassland | **0.01** | **0.11** | **0.22** |
| Total bird density | Forbs | **0.23** | **0.36** | **0.49** |
|  | WSG | −0.22 | −0.07 | 0.09 |
|  | Robel | −0.21 | −0.07 | 0.08 |
|  | Robel^2^ | **−0.24** | **−0.15** | **−0.07** |
|  | Ag | −0.21 | −0.08 | 0.06 |
|  | Grassland | **0.16** | **0.27** | **0.38** |
| SGCN | Forbs | −0.84 | −0.33 | 0.19 |
|  | WSG | −0.18 | 0.23 | 0.64 |
|  | Robel | **−0.93** | **−0.49** | **−0.04** |
|  | Robel^2^ | −0.13 | 0.16 | 0.45 |
|  | Ag | −0.66 | −0.27 | 0.12 |
|  | Grassland | **0.03** | **0.39** | **0.75** |
| Red-winged blackbird | Forbs | **0.36** | **0.66** | **0.96** |
|  | WSG | **−1.49** | **−0.85** | **−0.22** |
|  | Robel | −0.28 | 0.07 | 0.43 |
|  | Robel^2^ | **−0.84** | **−0.56** | **−0.27** |
|  | Ag | −0.44 | −0.05 | 0.34 |
|  | Grassland | **0.2** | **0.54** | **0.88** |
| Song sparrow | Forbs | −0.13 | 0.23 | 0.59 |
|  | WSG | −0.84 | −0.33 | 0.17 |
|  | Robel | **0.07** | **0.45** | **0.84** |
|  | Robel^2^ | **−0.63** | **−0.36** | **−0.09** |
|  | Ag | −0.21 | 0.18 | 0.57 |
|  | Grassland | −0.49 | −0.11 | 0.26 |
| Common yellowthroat | Forbs | −0.15 | 0.21 | 0.57 |
|  | WSG | **−1.53** | **−0.97** | **−0.41** |
|  | Robel | **0.12** | **0.6** | **1.08** |
|  | Robel^2^ | **−1.05** | **−0.68** | **−0.32** |
|  | Ag | −0.48 | −0.14 | 0.21 |
|  | Grassland | **0.1** | **0.31** | **0.52** |
| Dickcissel | Forbs | −1.57 | −0.5 | 0.57 |
|  | WSG | −0.09 | 0.63 | 1.36 |
|  | Robel | −1.63 | −0.77 | 0.08 |
|  | Robel^2^ | −0.52 | −0.16 | 0.2 |
|  | Ag | −0.12 | 0.45 | 1.02 |
|  | Grassland | **0.32** | **0.85** | **1.39** |
